# Determination of Fluorine by Ion-Selective Electrode and High-Resolution Continuum Source Graphite Furnace Molecular Absorption Spectrometry with Respect to Animal Feed Safety

**DOI:** 10.3390/ma17122812

**Published:** 2024-06-09

**Authors:** Zofia Kowalewska, Karolina Goluch, Waldemar Korol, Rafał Olchowski, Ryszard Dobrowolski

**Affiliations:** 1 Faculty of Civil Engineering, Mechanics and Petrochemistry, Warsaw University of Technology, Łukasiewicza 17, 09-410 Plock, Poland; 2 National Laboratory for Feedingstuffs, National Research Institute of Animal Production, Chmielna 2 Str., 20-079 Lublin, Poland; karolina.goluch@iz.edu.pl (K.G.); korol@clpp.lublin.pl (W.K.); 3 Department of Pharmacology, Toxicology and Environmental Protection, Faculty of Veterinary Medicine, University of Life Sciences, Akademicka Sq. 12, 20-950 Lublin, Poland; rafal.olchowski@up.lublin.pl; 4 Department of Analytical Chemistry, Institute of Chemical Sciences, Faculty of Chemistry, Maria Curie-Sklodowska University, M. C. Sklodowska Sq. 3, 20-031 Lublin, Poland

**Keywords:** fluorine determination, fluorides, ion selective electrode, ISE, potentiometry, HR-CS, molecular absorption spectrometry, graphite furnace, GaF, animal feed, calcium phosphate

## Abstract

Fluorine, depending on its concentration and chemical form, is essential or toxic to humans and animals. Therefore, it is crucial to be able to determine it reliably. In this study, fluorine was determined in animal feed after extraction with HCl (gastric juice simulation). The standard potentiometric method with a fluoride-selective electrode (ISE) and newly developed high-resolution continuum source graphite furnace molecular absorption spectrometry (HR-CS GFMAS) method was applied. Feed samples turned out to be a challenge for HR-CS GFMAS. Chemical interferences (formation of competing molecules, CaF, GaCl, and GaP, instead of the target GaF molecule) and spectral effects (including a phosphorous molecule spectrum and atomic lines) were identified. An additional difficulty was caused by reagent contamination with F and memory effects. Difficulties were eliminated/reduced. The quality of ISE analysis was multi-directionally verified (including comprehensive proficiency testing). A risk of inaccuracy at low F concentration, where the calibration relationship is nonlinear, was investigated. The results of both methods were consistent, which confirms the accuracy of the methods and informs that the extracted fluorine is in fluoride form. The results of extensive ISE tests conducted in Poland in 2021–2023 have shown that, in most cases, the fluoride content is significantly lower than the threshold values.

## 1. Introduction

Fluorine is an element commonly found in the environment. In terms of occurrence, it is the 13th most abundant element in the earth’s crust (0.059% by weight) [1]. Important minerals of fluorine are fluorapatite, fluorite, and cryolite [1,2,3].

Fluorine enters the secondary environment naturally through the weathering of rocks and minerals, volcanic gas emissions, forest fires, and, to a small extent, marine aerosols. Its anthropogenic sources include coal-fired energy, the ceramics industry, aluminum processing, and the production of phosphorus fertilizers. Agriculture also contributes to fluorine emissions from the use of phosphate fertilizers, sewage sludges, and pesticides [1,2,3].

In appropriate doses, fluorine is considered an element necessary for the proper development of animals and humans [1,4,5]. Deficiency of this element in the diet causes tooth decay, weakening of tooth enamel, and growth retardation, while its excess can lead to fluorosis, neurological deficits, worsening reproduction ability, as well as impaired thyroid and endocrine system functions [1,4,5]. Young animals are more susceptible to fluorosis because growing bones absorb fluoride more easily.

Animals absorb fluorine mainly as fluoride anions from feed and drinking water [1,4,5,6]. Typically, fluorine in tap water is of natural origin. The concentration of F is higher in groundwater, which is drawn from areas where rocks are rich in fluorine. Phosphate fertilizers may also contribute to excessive water pollution with F [2,3]. In other areas, tap water may be fluorinated to compensate for low fluorine levels. Fluorination is one of the most controversial topics in medicine, and some countries have decreased fluorine doses [1].

Plants can absorb F from the air through their leaves or from soil. The bioavailability of F in soil is usually low, but it depends on the plant species, the form of fluorine, and the soil type. Fluorine absorbed by plant roots is mostly retained there and does not reach the plant’s above-ground parts [1]. A bigger problem in animal nutrition may be feed contaminated with soil with high fluorine content or animals grazing [1,4]. However, it is believed that fluorine from the atmosphere leaches into deeper soil layers, and therefore, surface soils have a lower F content than underground soils. The presence of native F in soils depends on its content in the parent rock. Agricultural soils can be high in F when phosphorus fertilizers are used [3]. The cause of excessive fluorine consumption by farm animals may also be excessive supplementation with phosphate rock, the mineral fluorapatite. For this reason, the supplement can be purified of F before application [1].

Fluorine in animals accumulates mainly in calcified tissues that humans do not normally consume. Reducing and controlling F in feed is primarily aimed at protecting animal health. Therefore, the European Union has established permissible levels of fluorine content in animal feed [7]. The limits depend on the feed material. The threshold value for most animal feed is 150 mg kg^−1^. The threshold values for some materials, such as phosphate feed or krill, are much higher (2000 and 3000 mg kg^−1^, respectively). In Europe, fluorine content limits in animal feed are generally not exceeded [4]. However, fluorine emissions into the biosphere are constantly increasing due to the widespread use of fluorine compounds [1,2,5]. Therefore, the content of this element in animal feed should be monitored.

Numerous methods have been applied for F determination, i.e., wet chemical (including titrimetric), electrochemical (including potentiometry and voltammetry), spectrophotometric, chromatographic (including ion chromatography, gas chromatography, and capillary electrophoresis) methods, as well as neutron activation analysis and total reflection X-ray fluorescence [8,9,10,11,12,13]. Due to the extreme properties of fluorine, the conventional analytical approach for element determination, the application of atomic spectrometry techniques, is impossible or very rare; instead, indirect methods are developed [11,12,13].

In the European Union, a standard method for determining fluorine in animal feedstuffs relies on potentiometric determination with a fluoride-selective electrode (ISE) following extraction using 1 M hydrochloric acid [14]. Thus, the determined form of fluorine is free fluoride anions. Various methods can be used to carry fluorides into solution (e.g., open ashing, alkali fusion, microwave-induced combustion, and pyrohydrolysis [8,9,15,16]). However, the simple usage of 1 M HCl is fully justified in the case of animal feed analysis as it simulates gastric juices [17].

A special electrode made of rare earth fluorides was used to sense fluoride activity in a solution for the first time in 1966 [18]. The method is widely applied, and it is still being investigated. For example, it was used for the analysis of various seeds [16], and it is under investigation as an official method for fluoride control in infant formula [19]. It was pointed out that the ISE electrode is highly selective to fluoride anion over other anions and works over the range of five orders of magnitude of fluoride anion concentration [18]. Unfortunately, the calibration curve is nonlinear for lower fluoride content, which worsens with long electrode’s lifetimes [15]. The accuracy of the analysis can depend on the calibration procedure. The least accurate and most significantly underestimated results were obtained using a double standard addition method [20]. The ISE method needs to maintain pH in a narrow range, 5.2–5.5, to avoid OH^−^ interferences or transformation of fluoride anions into forms that are not detected by the method (HF or HF_2_^−^) [21,22]. Interferences can also come from cations (such as Fe^3+^ or Al^3+^), which can react with fluoride anions and form complexes, depending on pH [22]. To overcome interference problems and maintain ionic strength, sodium acetate and sodium citrate are often added to the measured solution [15,17,22,23]. The effectiveness of various masking agents has been investigated lately, and the best results have been obtained for TISAB (total ionic strength adjustment buffer) [24]. Inconsistencies in the results of determining fluorides in tea infusions using ISE and ion chromatography methods were attributed to the difficulty of complete isolation of the fluoride ion signal in ion chromatography [22]. On the other hand, the good performance of citrates as the complexing agent was confirmed [22].

Currently, one of the more modern analytical techniques available is graphite furnace high-resolution continuum source molecular absorption spectrometry (HR-CS GFMAS). The technique was introduced in the first decade of the XXI century [8,25,26,27], and it has been widely developed in the second decade up to now [11,12]. The HR-CS GFMAS relies on generating a target molecule of an analyte in a heated graphite furnace. The appearing molecules absorb the radiation of a high-intensity xenon lamp at specific wavelengths, which is measured by a high-resolution system. To date, HR-CS GFMAS has not been used for the determination of fluorine in animal feed.

The goal of this work was to determine fluorine in animal feed using ISE and HR-CS GFMAS methods. In both cases, for the sample preparation step, the extraction with 1 M HCl, according to European Standard EN 16279:2012 [14], was selected. The same sample preparation procedure eliminates doubts connected with the efficiency of extraction.

## 2. Materials and Methods

### 2.1. Instrumentation and Its Operation

Fluoride measurement in the solutions by the ISE method was made with a combined Orion fluoride electrode (Thermo Fisher Scientific, Waltham, MA, USA) and a pH-meter CG 843 P (Schott, Hofheim, Germany). Each solution was mixed on a magnetic stirrer (Wigo, Pruszków, Poland). 

A high-resolution continuum source absorption spectrometer contrAA 800 (Analytik Jena, Jena, Germany) was used to determine fluorine via molecular absorption. The radiation source was a high-intensity xenon lamp working in a hot spot mode and emitting continuum radiation. A double monochromator comprising a prism and an echelle grating enabled it to reach an excellent resolution of 1.4 pm per pixel. Of the 588 pixels of the CCD detector, 200 were available and covered the measured spectral range (211.1072–211.3874 nm). The range was centered at 211.248 nm, the center of the most sensitive rotational line of the target molecule, gallium monofluoride (GaF). The line belongs to the 0 → 0 vibrational transition within the X^1^Σ → C^1^Π electronic transition of this molecule. Seven pixels were selected for GaF molecule absorption measurements. An iterative mode, based on absorption minima (IBC mode), was used for background correction.

The gallium monofluoride was generated in a graphite furnace with an integrated PIN platform. Each new graphite furnace was initially covered with 230 µg of zirconium (three 25 µL and two 20 µL injections of 0.2% Zr solution, each followed by thermal pretreatment according to a routine heating cycle [28]). 

Additionally, for the thermal stabilization of fluorine, a mixture of palladium and zirconium (5 µL of a solution containing 0.1% of Pd and 0.003% of Zr) was injected before the start of each graphite furnace heating cycle. This first injection also consisted of 6 µL of a 0.5% Ga solution. The heating program of graphite furnace is presented in Table 1. For thermal reduction of Pd, the temperature of 1100 °C was applied in step 5. The furnace was then cooled, and a second injection was executed involving 6 μL of 0.5% Ga solution, 5 μL of 0.4% sodium acetate (NaAc solution), and 6 μL of working solution. The role of NaAc was the neutralization of possibly formed volatile HF. 

### 2.2. Reagents, Standards, and Solutions

Sodium fluoride at a concentration of 1000 mg L^−1^ (Merck, Darmstadt, Germany) was used as a fluorine stock standard solution. 

The following reagents were used in the ISE method and for sample preparation: hydrochloric acid 1 M was prepared by dilution of hydrochloric acid 35–38% (Avantor Performance Materials Poland S.A., Gliwice, Poland). The acetate buffer was prepared from sodium acetate trihydrate (Eurochem, Tarnów, Poland). Its pH was fixed by acetic acid 99.5–99.9% (Avantor Performance Materials Poland S.A., Gliwice, Poland) at 7.0. Citrate buffer was prepared from trisodium citrate dihydrate (Chempur, Piekary Śląskie, Poland) and perchloric acid 70% (Chempur, Piekary Śląskie, Poland). Distilled water was used to prepare all the solutions. In interference studies, 1000 mg L^−1^ standards of Fe, Al, and Ca (from Merck, Darmstadt, Germany) were used.

The following reagents were used in the HR-CS FMAS method: 99.999% gallium (III) nitrate (V) hydrate (from Sigma-Aldrich, Saint Louis, MO, USA), 10 g L^−1^ palladium modifier (from Merk, Germany), 2 g L^−1^ zirconium standard solution (the fix anal was diluted in 500 mL of water; from Sigma Aldrich Chemie GmbH, Steinheim, Germany), 5% ammonium dihydrogen phosphate (V) solution in water (a modifier for AAS form Teknolab, Drobak, Norway), 0.1% P standard as phosphoric (V) acid (from CPAchem, Bogomilowo, Bulgaria), 0.1% Ca solution in 2.5% nitric (V) acid (a Ca standard form GUM, Warsaw, Poland), 35–37% hydrochloric acid from Chempur, Piekary Śląskie, Poland) and sodium acetate (from VHR BDU Chemicals, Leuven, Belgium). Water, purified in a reverse osmosis process, was used in HR-CS GFMAS experiments. 

The used reagents were at least of analytical grade.

### 2.3. Samples and Their Preparation

Fluorine was extracted from samples with 1 M hydrochloric acid according to the EN 16279 standard method [14]. In the ISE method, 0.5 g of sample was weighed into a plastic beaker. Moreover, 20 mL of 1 M HCl was added, and extraction was conducted for 20 min at room temperature (23 °C ± 1 °C). To ensure appropriate pH (5.5 ± 0.1) and ionic strength, 50 mL of acetate buffer and citrate buffer were added. The solutions were transferred to a 200 mL plastic flask and brought to volume with distilled water. The solutions were filtered before analysis. Most samples (exceptions could be made to calcium phosphate samples) were analyzed on the day of solution preparation.

The working calibration solutions were also prepared according to the EN 16279 standard method. The used calibration range covered 0.01–10 mg L^−1^ F content. All working solutions contained 1 M HCl, diluted 1:10 *v*:*v* (i.e., 0.1 M HCl), as well as acetate buffer and citrated buffer at the same concentration as samples. Measurements were performed from the lowest standard concentrations to the highest. 

In the HR-CS GFMAS method, the initial dilution ratio was the same, 1:40 *m*:*v*, as in the ISE method (extraction from 1.25 g of a sample to 50 mL of the volume of solution). Due to organizational reasons, the HR-CS GFMAS analysis was performed at least a few days after the extraction. 

Further pretreatment for HR-CS GFMAS depended on the solution’s appearance and expected F content as follows:

– Solutions of calcium phosphate (V) were opalescent, but no sediment precipitation (even a few weeks after preparation) was observed; the solutions were diluted in the ratio 1:4000 or 1:20,000 *m*:*v*;
– Solutions of other samples were inhomogeneous; after intensive shaking, the precipitated sediment was separated by filtration; the filtrates were further diluted, and the dilution ratio depended on F content.

The dilution ratio of a single dilution step was not higher than 10. Thus, some dilutions comprise a few steps. For dilution and preparation of solutions for standard addition calibration, 1.5-volume Eppendorf vials were used.

In the standard addition method, the following two kinds of solutions were prepared:

– Type A: an extract from a given sample at a given dilution ratio;
– Type B: an extract from a given sample, like solution A, but with the addition of 0.1 mg L^−1^ of F.

In this study, especially, the following three samples were investigated:

– S1, calcium phosphate, containing a high concentration of F (approximately 1700 mg kg^−1^),
– S2, a mixed feed for turkey, which represents samples with low content of F (approximately 12 mg kg^−1^ of F),
– S3, a spiked feed for chickens, representing samples with moderate F content (approximately 70 mg kg^−1^ of F).

## 3. Results and Discussion

### 3.1. Studies Using the Ion-Selective Electrode Method

Two calibration techniques were used for fluoride determination depending on the fluoride content of the sample, i.e., the interpolation technique when the concentration was within the nonlinear range of the curve and the standard addition technique when the concentration was within the linear range. An example of the calibration curve in the range of 0.01–10 mg L^−1^ is presented in Figure S1. Good linearity is achieved in the range 0.1–10 mg L^−1^ (the determination coefficient R^2^ equals 1.0000), while at lower concentrations of fluorides, the calibration line curves (the determination coefficient R^2^ for the 0.01–10 mg L^−1^ range equals 0.9925).

As recommended by the EN 16297 standard [14], the standard addition method involves adding 5 mL of a working standard to the sample solution. The concentration of the standard is adjusted to cause a potential drop of 5 to 40 mV. For example, in the case of a spiked feed containing approximately 70 mg kg^−1^ of fluorides in the initial sample, the added standard contained 3 mg L^−1^ of fluorides, and in the case of a phosphate sample containing approximately 1700 mg kg^−1^ of fluorides, the added standard contained 100 mg L^−1^ of fluorides.

In the standard addition method, the fluoride content was calculated using the following formula:

$$c_{s}=c_{A}\frac{0.1}{1.1\times 10^{\frac{\Delta E}{S}}-1}$$

c_s_—fluoride concentration in the sample solution, mg L^−1^,

c_A_—fluoride concentration of the added standard solution, mg L^−1^,

ΔE—potential difference (mV) after adding 5 mL of standard solution,

S—the slope of the calibration curve in the linear range [mV].

If the entire calibration range was utilized and the quadratic approximation of the calibration curve applied, the determination coefficient R^2^ was equal to 0.9988. 

The error of using linear calibration in the low concentration range was evaluated. For a sample containing 13.2 mg kg^−1^ of fluorides (approximately 0.03 mg L^−1^ in solution), the results of fluoride determination using linear fitting of the calibration curve would be 13.3 mg kg^−1^. Thus, the difference in this case is slight (a relative difference of less than 1%). Unfortunately, at lower fluoride concentrations (e.g., 4 mg kg^−1^, i.e., 0.01 mg L^−1^ in solution), using linear calibration would lead to significant result overestimation (i.e., the result of 5.2 mg kg^−1^, i.e., 0.013 mg L^−1^ in solution and a relative difference of 30%).

The linear fitting of the calibration curve was applied for fluoride concentrations of 0.1 mg L^−1^ and more.

For the evaluation of the detection and determination limits, a sample with a low content of fluorides (a mixed feed for turkey) was analyzed seven times. At the average result of fluoride concentration equal to 4.81 mg kg^−1^, the standard deviation was 0.10 mg kg^−1^, and the relative standard deviation was 2.2%. The detection and determination limits, calculated as three and ten times the standard deviation, amounted to 0.3 and 1.0 mg kg^−1^, respectively. The values are satisfactory from the point of view of the aim of this work.

The precision of the analysis was evaluated based on the differences between parallel results for samples with low or high fluoride content. The results of the analyses are presented in Tables S1 and S2, respectively. The fluoride content in the first group’s samples was from 5.0 up to 6.1 mg kg^−1^. The average result was 5.4 mg kg^−1^, and the average difference was 0.04 mg kg^−1^ (relative absolute value of difference: 3.7%). The content of fluorides in the samples of the second group was from 1348 up to 4353 mg kg^−1^. The average result was 2262 mg kg^−1^, and the average difference was 28 mg kg^−1^ (average relative absolute value of differences: 2.9%). The precision is satisfactory.

For evaluating accuracy, a known amount of fluorides was added (as a solution) to a sample before extraction and recovery of analysis were determined. The recovery was defined as the ratio of the obtained result to the calculated (theoretical) result, expressed in percent. For calcium phosphate, the results of recovery evaluation were as follows: 109, 104, 106, 95, and 102% (103% on average). For mixed feed for turkey, the recovery was as follows: 81, 87, and 79% (82% on average).

**Figure 1:**
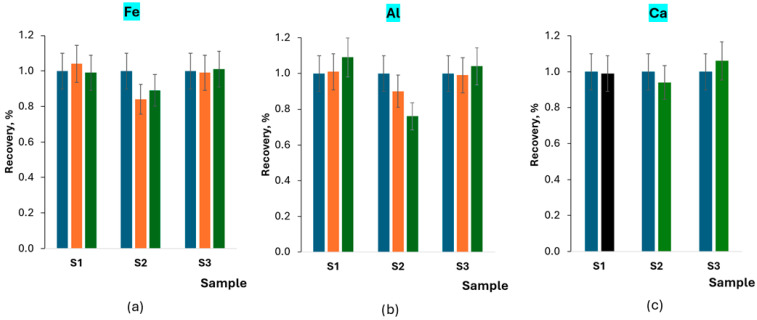
Influence of potential interferents on recovery of fluorine contained in samples S1, S2, and S3: Fe (**a**), Al (**b**) and Ca (**c**).

Further recovery experiments were conducted but with the addition of potential interferents. Fluoride anions can form complex compounds with Fe^3+^ and Al^3+^ [17,22,23,24]. It is expected that the reactions do not occur in the presence of citrates. Therefore, the EN 16279 method [14] was designed to use the masking agent. A relatively large amount of potential interferents was added to the extraction solution to verify the lack of Fe and Al effects. The effect of additional calcium concentration was also studied, first of all to evaluate its influence on the extraction efficiency. The results of the investigation of the three samples mentioned in Section 2.3 are presented in Figure 1.

It can be concluded that Fe, Al, and Ca do not influence analysis results for samples S1 and S3, analyzed using the standard addition method and containing quite a high amount of fluorides. Some tendency to decrease results (up to 20%) can be observed in the case of Fe and Al added to sample S2. It can be caused by the consumption of some part of fluoride anions in complexation reactions or sample matrix influence, changing the slope of the calibration curve (which is not compensated with the standard addition method).

Proficiency testing results were used to evaluate data quality. In Table 2, the results of 13 proficiency testing programs devoted to the determination of fluorides in animal feed are presented. The investigations were carried out over the last 17 years. Various materials were investigated with various fluoride content (assigned values from 10 up to 1800 mg kg^−1^). As the obtained z-score values show, the results of analyses of the National Laboratory for Feedingstuffs in Poland are satisfactory. The ISE method, as described in the current study, was applied.

It is essential to add that a relatively low number of laboratories participated in the proficiency testing (4–13), and almost all results were obtained using the same ISE method. Thus, the proficiency of laboratories was confirmed in the frame of the same analytical technique. Unfortunately, in the case of a systematic method error, it was not possible to reveal the error. Therefore, it can be concluded that accuracy was not entirely verified.

### 3.2. Studies Using High-Resolution Continuum Source Graphite Furnace Molecular Absorption Spectrometry Method

#### 3.2.1. Selection of the Target Molecule

According to the literature [11,12,25,26,27,29,30,31,32,33,34,35,36,37,38,39,40], a few molecules, monofluorides of elements of IInd and XIIIth periodic table groups, were synthesized in the graphite furnace to determine fluorine via molecular absorption spectrometry. Some of such research was carried out in the past using classical AAS equipment of medium resolution [26,27,40], but real progress and wide application started with the introduction of high-resolution continuum source apparatus [11,12,25,26,27,29,30,31,32,33,34,35,36,37,38,39]. The device enables access to various wavelengths and efficient background correction, among others [25,26,27].

The HR-CS GFMAS enables the determination of the total amount of fluorine if only initial fluorine compounds are converted into the target monofluoride.

Using HR-CS GFMAS, gallium monofluoride [29,30,31,32,33,34] or calcium monofluoride [35,36,37] was mainly applied as a target molecule. The most crucial advantage of GaF is excellent sensitivity and low detection limits (characteristic mass of 7 pg and detection limit of 5 pg [30]); however, it is achieved using a complicated chemical modification. For the last reason, the application of another molecule, CaF, found popularity. Unfortunately, the sensitivity of such analysis was one to two orders of magnitude lower than in the case of GaF [35,36,37].

The conclusion on the relative sensitivity of F determination via absorption of GaF and CaF molecules in HR-CS GFMAS does not agree with the results of an alternative technique, high-resolution continuum source molecular spectrometry with flame as a chemical reactor. Careful adjustment of the measured spectral range and summing signals of many available pixels of the target molecule enabled obtaining similar (or even better) sensitivity and detectability for CaF [41] (and SrF [42]) as for GaF [13].

For the purpose of this work, it was decided to select GaF. It was expected that it would ensure achieving good sensitivity and detectability (as in the literature on HR-CS GFMAS). Therefore, it would allow sample dilution to decrease the content of the matrix in the graphite furnace. Another important reason is that animal feed could contain high and variable calcium concentrations that would result in variable sensitivity of F determination. On the other hand, animal feed is not expected to contain a significant amount of gallium. Additional difficulty could occur due to HCl application. It was found that a spectrum of CaCl, a molecule that can be formed if a measured solution contains HCl, overlaps the spectrum of the CaF molecule [37].

#### 3.2.2. Selection of Spectral Conditions and Analytical Range

For the measurement of GaF absorption, the most sensitive line at 211.248 nm has been selected as in most of the previous papers [29,30,31,32,33,34]. This is the only single line of GaF molecule that appears in the measured spectral range. The line resembles common atomic single lines (used in AAS). As the line is relatively broad, seven pixels have been selected for F measurements. Iterative background correction designed for atomic absorption measurements has been used.

It is interesting that in the measured spectral range, a gallium atomic line is visible (211.197 nm, relative sensitivity 0.026). The appearance of this line indicates the presence of unbonded gallium in the graphite furnace, which is a good symptom (excess gallium is needed). The Ga 211.197 nm line and the 211.248 nm GaF line lie at a significant and “safe” distance from each other (do not overlap).

#### 3.2.3. Pyrolysis/Vaporization Curves

The essential tool to recognize the behavior of an analyte in a graphite furnace is a series of measurements of an investigated solution with the stepwise increase of pyrolysis step temperature or measurement (i.e., vaporization) step temperature. In this way, respectively, pyrolysis or vaporization curves are obtained. Such experiments were performed for 0.1 mg L^−1^ standard solution and 0.1 mg L^−1^ standard solution with the addition of HCl, as well as solutions of samples S1–S3.

**Figure 2:**
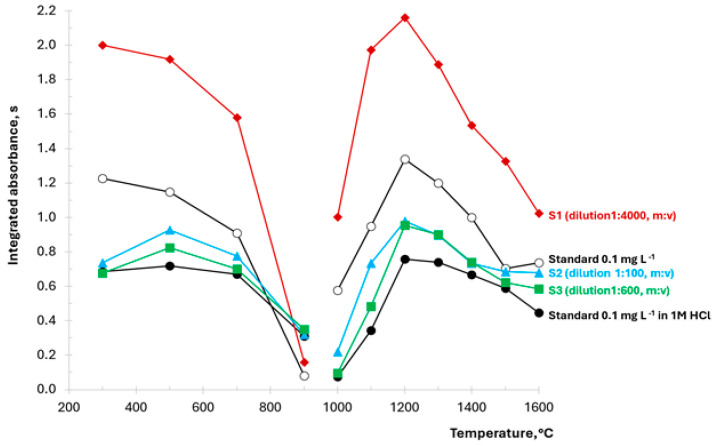
Pyrolysis–vaporization curves obtained for solutions of samples S1–S3 and 0.1 mg L^−1^ standard in a pure solution or as a solution containing 1 M of HCl. For better visibility, all absorbances for sample S1 were divided by two.

The results are presented in Figure 2. All pyrolysis curves were obtained using the 1300 °C temperature of the vaporization step (step number 12), and all vaporization curves were obtained using the 500 °C temperature of the pyrolysis step (step number 10).

Generally, the course of the curves for the five investigated solutions is quite similar. Significant fluorine losses certainly occur at pyrolysis temperatures above 700 °C. In the case of the standard solution of 0.1 mg L^−1^ and the highly diluted sample S1, the losses seem to occur even at lower temperatures. However, for these two solutions, the signals for pyrolysis temperatures of 300 °C and 500 °C are quite similar, taking into account approximately 10% relative difference of results of parallel measurements at given conditions.

Considering vaporization curves, the maximum signal is obtained for all investigated solutions using the 1200 °C temperature of step 12. The signal increase with the temperature from 1000 to 1200 °C is sharp, while the decrease of the signal at the temperature above 1200 °C is slower. It seems that the presence of HCl in the 0.1 mg L^−1^ standard solution or in the S2 and S3 sample matrix could slow down the signal decrease. Based on the pyrolysis–vaporization curves, the selected temperatures would be 500 °C for the pyrolysis step and 1300 °C for the vaporization step.

**Figure 3:**
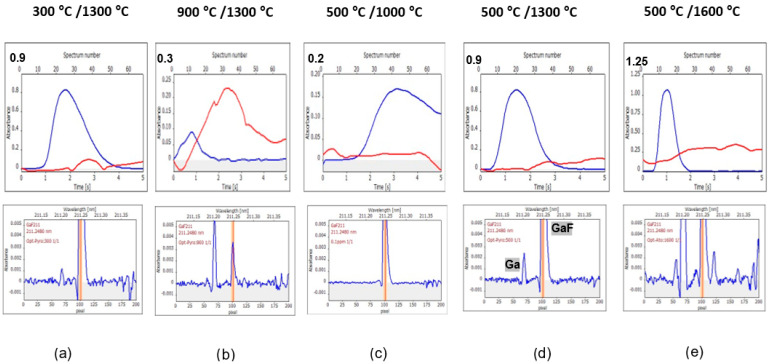
Signals of a 0.1 mg L^−1^ solution of fluorine as NaF, obtained using the following pyrolysis/vaporization temperatures: (**a**) 300 °C/1300 °C, (**b**) 900 °C/1300 °C, (**c**) 500 °C/1000 °C, (**d**) 500 °C/1300 °C, and (**e**) 500 °C/1600 °C. The main figure of each subfigure presents absorbance–time changes (the blue line is the analyte line, and the red line is the background line). The smaller figure of each subfigure denotes an enlarged wavelength-resolved absorption spectrum. The vertical orange line denotes the central pixel of the number 101.

**Figure 4:**
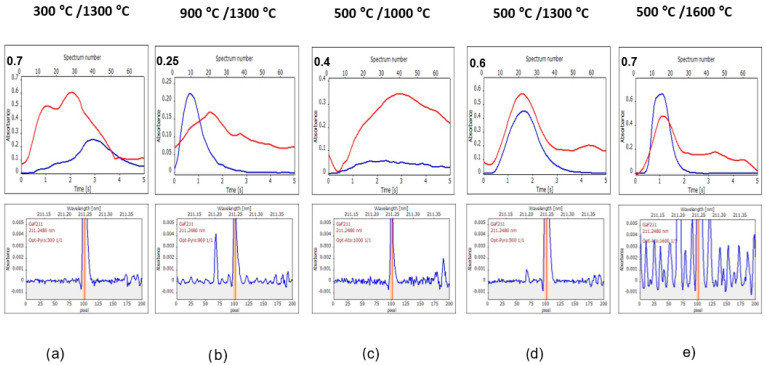
Signals of a solution of sample S2 (dilution 1:100 *m*:*v*, 1 M HCl), obtained using the following pyrolysis/vaporization temperatures: (**a**) 300 °C/1300 °C, (**b**) 900 °C/1300 °C, (**c**) 500 °C/1000 °C, (**d**) 500 °C/1300 °C, and (**e**) 500 °C/1600 °C. The main figure of each subfigure presents absorbance–time changes (the blue line is the analyte line, and the red line is the background line). The smaller figure of each subfigure denotes an enlarged wavelength-resolved absorption spectrum. The vertical orange line denotes the central pixel of the number 101.

To better understand the processes in a graphite furnace, the signals measured using selected pyrolysis/vaporization temperatures are presented for 0.1 mg L^−1^ of F standard solution (Figure 3), S2 sample solution (Figure 4), S1 sample solution (Figure S2), S3 sample solution (Figure S3), and 0.1 mg L^−1^ of F standard solution in 1 M HCl (Figure S4). Each figure contains upper subfigures with time-resolved absorption (blue lines of GaF signal and red lines of BG signal) and lower subfigures with wavelength-resolved absorption in the measured spectral range. The lower situated, wavelength-resolved signals are presented at an enlarged scale to observe better baseline changes and the appearance of new phenomena (the result of this is cutting off the top of the high GaF peak). The scale of Y-axes in the upper subfigures is software-selected and varies, depending on the height of the signal. In contrast, the scale of Y-axes in lower figures was adjusted to always be the same for better comparison. The center of each wavelength-resolved absorbance picture is always the GaF molecular absorption line, while the Ga atomic absorption line has a maximum at pixel number 68 if it appears at all. The two absorption signals are exemplary and labeled in one of the subfigures of Figure 3.

Figure 3, Figure 4 and Figures S2–S4 show the following:

– Similarity of registered curves for 0.1 mg L^−1^ F standard and S1 highly diluted sample.
– Similarity of registered curves for 0.1 mg L^−1^ F with HCl and less diluted samples S2 and S3.
– One of the significant differences between these two groups is the higher background (BG) for the second group, especially distinctly seen for measurements using 300 °C/1300 °C and 500 °C/1000 °C.
– Another difference is a higher signal of Ga for the solutions of the first group (it is seen, for example, when Figure 2 and Figure S4 are compared, obtained for 0.1 mg L^−1^ of F and 0.1 mg L^−1^ of F with addition of 1 M HCl, respectively).
– For all the five investigated solutions, the same tendency of the GaF signal changes (appearance and shape) is observed with the changing temperatures; as is seen in the upper subfigures, with the increase of both pyrolysis and vaporization temperatures, the GaF signals move to the beginning of the measurement step and become narrow.
– Other phenomena appearing with the increase of both pyrolysis and vaporization temperatures are as follows: an increase of the Ga signal at 68 pixel and the appearance of a new structure background; the last phenomenon takes place especially using 1600 °C vaporization temperature; and the structure background observed using high 1600 °C vaporization temperature is a dense bush of lines in the case of samples S2 and S1 (Figure 4 and Figure S2, respectively).

The observed phenomena indicate interferences, which will be further considered in the following sections.

#### 3.2.4. Influence of Hydrochloric Acid

Hydrochloric acid is indispensable in the applied extraction procedure [14]. It simulates the composition of the stomach environment and does not disturb potentiometric measurements. However, as was shown above, it is a cause of a background (time-resolved subfigures of Figure 3, Figure 4 and Figures S2–S4). 

**Figure 5:**
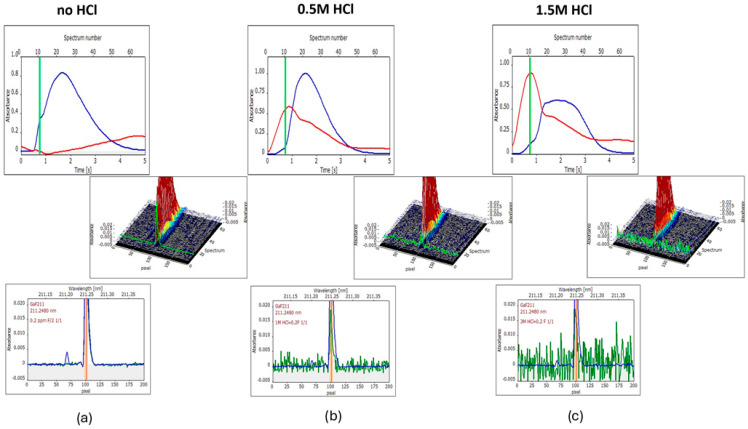
Influence of increased amount of HCl in solution on integrated absorbance and background: (**a**) no HCl, (**b**) 0.5 M HCl, and (**c**) 1.5 M HCl. Pyrolysis/vaporization temperatures: 500 °C/1300 °C. In middle parts of figures (**a**–**c**), 3-dimisional spectra are presented (wavelength- and time-resolved absorption), while the upper and lower subfigures contain 2-dimensional pictures, time-resolved absorption and wavelength-resolved absorption. The wavelength-resolved green signals were obtained at spectrum number 11 (time 0.8 s), which is marked in time-resolved spectra as a vertical light-green line.

To confirm the HCl effect, a series of solutions with the same content of F and the increasing content of HCl was measured. As can be seen in Figure 5, the HCl effect can be connected with the appearance of an intensive background at the beginning of the vaporization curve (red curves at the time-resolved absorbance subfigures with maxima near the 11th spectrum, i.e., 0.7–0.8 s). The same BG is seen in green in the three-subfigures (wavelength- and time-resolved absorption) and the wavelength-resolved absorbance subfigures (the green signals measured only at the 11th spectrum overlap the blue signals, which is an average spectrum (averaging throughout the 5-s vaporization phase). A higher amount of HCl can change the shape of the GaF signal, which is shown in the time-resolved subfigure of Figure 4c. Also, a decrease of integrated absorbance takes place by approximately 10% and 30% for 0.5 M HCl and 1.5 MHCl, respectively, relative to the measurement without HCl.

The conclusions from the experiments are as follows:

– The HCl presence in the measured solution is a cause of the background appearing early in the vaporization step, which is separated in time from the GaF peak; at the highest concentration of HCl in a solution of a sample, 1 M, the spectral effect is some disturbance of baseline, which seems to be insignificant if Figure 3d and Figure S4d are compared (the selected pyrolysis/vaporization temperatures are equal to 500 °C/1300 °C).
– Due to the presence of HCl in the measured solution, a decrease of the Ga peak (comparison of Figure 3, Figures S4 and S5) and a decrease of the GaF signal occur. It can be explained as the formation of a concurrent compound, GaCl. The bonding energy of GaCl of 481 kJ mol^−1^ is relatively high, even though the bonding energy of GaF is 577 kJ mol^−1^ [43]. Therefore, less Ga is available for the formation of the target molecule, GaF, which would cause a decrease in the accuracy of the measurement and underestimation of F.

#### 3.2.5. Other Interferences

The spectra presented in Figure 3, Figure 4 and Figures S2–S4 contain additional signals, apart from the Ga atomic line at 68 pixel and the GaF line. The additional signals are significantly increased when the graphite furnace temperature is higher, i.e., in the options of pyrolysis/vaporization temperature 900 °C/1300 °C and, especially, 500 °C/1600 °C. Additional lines are always observed, even in the measurements of simple solutions of the F standard (Figure 3). 

According to the software of the contrAA apparatus [28], at least 22 atomic lines and spectra of five molecules can be observed in the measured spectral range. The quoted atomic lines are of low sensitivity. These are mainly lines of elements requiring higher atomization temperatures (higher than 1600 °C). These elements are not expected to be present in a fluorine standard solution. No spectra of the six quoted molecules (CS, NO, OH, PO, and SiO) resemble the spectra obtained in this work. 

**Figure 6:**
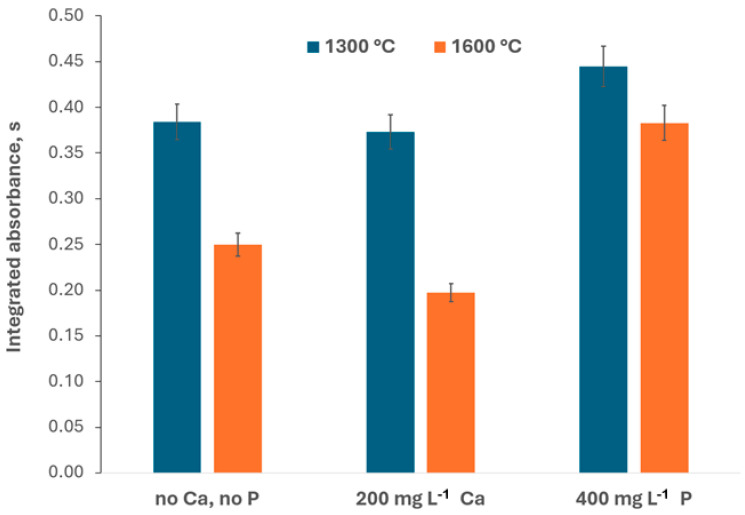
Signals of 0.1 mg L^−1^ of F, depending on the presence of 200 mg L^−1^ of Ca or 400 mg L^−1^ of P in the measured solution and the temperature of the measurement step.

In further experiments, model solutions containing 200 mg L^−1^ of Ca and 400 mg L^−1^ of P (approximately 1200 mg L^−1^ of PO4^3−^ ion, coming from NH_4_H_2_PO_4_) were investigated using 1300 °C or 1600 °C vaporization temperature (Figure 6). 

The Ca effect at the 1300 °C vaporization temperature is negligible (the recovery relative to the result of measurement of a solution without Ca is equal to 102%), but at the 1600 °C, a distinct decrease of the GaF signal occurs (recovery of 79%). Introducing 200 mg L^−1^ of Ca to the measured solution does not lead to new spectral events. The Ca effect at 1600 °C vaporization temperature is probably of a chemical nature. It is the consequence of the concurrent reaction of F with Ca (the bonding energy of CaF of 527 kJ mol^−1^ is not much lower in comparison with the bonding energy of GaF [43]). Probably, the effect does not occur at lower investigated vaporization temperatures because the temperatures are not high enough for the atomization of Ca.

As Figure 6 indicates, at the 1300 °C vaporization temperature, the presence of phosphate leads to a distinct increase in the measured GaF signal (recovery 122%). This effect is much higher using 1600 °C vaporization temperature (recovery 153%). It is interesting that in the spectra acquired in measurements of solutions containing NH_4_H_2_PO_4_, an intensive structure background appears that resembles the spectra with a bush of lines in Figure S2 and Figure 4, measured using 900 °C/1300 °C and 500 °C/1600 °C pyrolysis/vaporization temperatures. Figure S2 refers to a highly diluted (1:4000 *m*:*v*) solution of sample S1 (which is calcium phosphate used as animal feed), and Figure 4 refers to a low-diluted (1:100 *m*:*v*) solution of sample S2 (a feed for turkey). Various dilutions were applied due to the various fluorine contents. Nevertheless, the P content in the measured solutions was similar, approximately 50 mg L^−1^ in the S1 sample solution (calculation based on stoichiometry) and 70 mg L^−1^ in the S2 sample solution (calculations based on known P content in the original sample and the assumption of 100% extraction efficiency).

**Figure 7:**
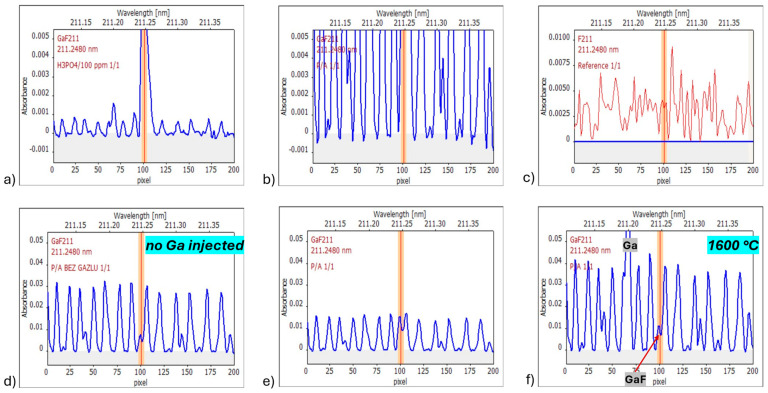
Wavelength-resolved absorbance in measurements of a solution of H_3_PO_4_ containing 32 mg L^−1^ of P (**a**) or a solution of NH_4_H_2_PO_4_ containing 400 mg L^−1^ of P (**b**,**d**–**f**). Subfigures (**b**,**d**) differ only in the y-scale. Measurement temperature: 1300 °C (**a**,**b**,**d**,**e**) or 1600 °C (**f**). In case (**d**), the gallium solution was not injected. Subfigure (**c**) comes from the Aspect CS database [28], where it was attributed to the PO molecule.

The next reagent was measured to verify the origin of the intensive “bush” background, a standard of P, H_3_PO_4_. Figure 7 shows spectra of H_3_PO_4_ (Figure 7a) and Na_4_H_2_PO_4_ (Figure 7b–d,f). A reference spectrum from a software database attributed to the PO molecule [28] is presented in Figure 7c. The scale of Figure 7b is the same as in Figure S2 and Figure 4. The spectrum of Na_4_H_2_PO_4_ in Figure 7b is very intensive. Therefore, Figure 7e was prepared with the same spectrum but at a reduced scale.

The following can be seen in Figure 7:

– Spectra of H_3_PO_4_ and NH_4_H_2_PO_4_ can be interpreted as the same “fingerprint spectrum”; the observed differences come from various relative intensities of the P molecule, the Ga atomic line (the 68th pixel), and the GaF molecule (the peak centered at the 101st pixel).
– The only spectrum measured at the higher vaporization temperature of 1600 °C (Figure 7f) shows (in comparison with Figure 7e measured at the 1300 °C vaporization temperature) much higher intensity of both the P-molecule spectrum and the Ga signal; on the other hand, the signal of GaF is less intensive (which corresponds well with Figure 2).
– The P-molecule spectrum directly overlaps the measured range of seven central pixels used for evaluation of the F signal.
– It is impossible to obtain a pure P-molecule spectrum because the graphite furnace is contaminated with fluorine; the contamination source is Ga salt, and the GaF signal for blank significantly decreases when the Ga solution is not injected (Figure 7d); some residual GaF signal still appears and makes it impossible to apply the LSBC (least squares background correction) method for elimination of interference by the P-molecule.
– The experiments illustrated by Figure 7d,e differ only in Ga solution application; the application of Ga in the experiment from Figure 7e causes an increase of GaF signal and a decrease of P-molecule signal; the Ga atoms could form the GaP molecule (bonding energy of 439 kJ mol^−1^ [43]).
– The spectra obtained for phosphates are similar to each other but differ from the reference spectrum attributed to the PO molecule [28]; there is literature data on the formation of the P_2_ molecule in the graphite furnace, apart from the PO molecule [44,45]; it is not sure which molecule is formed in the current research; it is undoubtedly another molecule than the one used for software spectrum generation.

The spectra obtained for samples S1 and S2 (Figure S2 and Figure 4, respectively) at vaporization temperature 1600 °C do not resemble exactly the P-molecule spectra from Figure 7. It seems that an additional effect occurs for the solutions investigated in Section 3.2.3 when a higher vaporization temperature of 1600 °C is applied. This is the atomization of an element/elements, resulting in their atomic lines’ appearance. As the lines also appear for a simple standard solution of F (Figure 3e), the atomic lines cannot be attributed to the sample matrix. Probably, these are low-intensity lines of Ga, which is always injected into GF to form GaF molecules. 

Thus, the wavelength-resolved spectra in Figure S2e and Figure 4e show the following:

– Overlapping signals of molecular absorption of GaF and a P-molecule.
– Signals of single atomic lines, including the Ga 211.2000 nm line (the 68th pixel).

However, the applied vaporization temperatures (even 1600 °C) are relatively low in terms of the temperature required for the atomization of most elements. It explains, for example, why the Fe signal is not seen (211.197 nm Fe line), although some investigated samples contain a lot of Fe. This Fe line was observed in flame experiments [13].

The registered atomic lines are distant from the wavelength range of absorbance by GaF and do not disturb the analyte signal directly. On the other hand, the overlap of the P-molecule spectrum on the GaF signal causes an overestimation of the results of F determination.

#### 3.2.6. Strategy for F Measurements and Figures of Merit

Interferences due to hydrochloric acid, calcium, and phosphates were stated in previous sections. An additional issue is the need to keep a balance in the gallium application. The amount of applied gallium should be high enough to form the GaF molecule efficiently. Unfortunately, the available gallium salt (gallium (III) nitrate of 99.999% purity) is contaminated with fluorine. These problems were taken into account in the selection of measurement conditions. 

The following was decided:

– To use the preliminary selected 1300 °C vaporization temperature; this temperature is high enough to form the GaF molecule efficiently (Figure 2), and some excess of Ga is visible as a 211.2000 nm atomic line at the 68th pixel in wavelength-resolved spectra of Figure 3d, Figure 4d and Figures S2d–S4d; simultaneously, the 1300 °C vaporization temperature is low enough to not cause atomization of calcium, which could bond fluorine and lead to underestimation of the F determination; also, at this temperature, the spectral effect of phosphates does not occur (lack of P-molecule spectra in Figure 4d and Figure S2d);
– To use standard addition calibration to overcome chemical interferences, the HCl effect and the phosphate effect, it is important to remember that animal feed can contain other possible interferents, which were not investigated here; standard addition calibration is a sure safeguard against such problems as well as a simple tool against differences in the physical properties of standards and sample solutions.
– To use a relatively high but accepted from the point of view of F level dilution ratio of initial solutions, it would diminish interferences; e.g., due to the high dilution of sample S1, being calcium phosphate, no P-molecule spectrum is visible in the wavelength-resolved spectrum in Figure S2d (the S1 sample is diluted 1:4000 *m*:*v* here); the high dilution of this kind of material containing a lot of F (above 1000 mg kg^−1^) justifies resignation from standard addition calibration for this kind of samples.

Taking into account these decisions and the conditions specified in Section 2.1, a calibration curve was obtained for the range of concentrations of 0.05–0.4 mg L^−1^. Satisfactory parameters of the curve are presented in Table 3. Excellent sensitivity was achieved, 2.1 pg (better than in published works [29,30,31,32,33,34]), which relates to concentration 0.0003 mg L^−1^ in the measured solution injected in the volume of 6 µL. However, due to contamination of Ga with F, the blank (0.030 mg L^−1^) is a few orders of magnitude higher. The instrumental detection limit of 0.01 mg L^−1^ is satisfactory for the goal of this work.

The detection/determination parameters in the analysis of real samples are specified in Table 4. All samples were divided into two groups according to the expected F content and threshold F concentration. 

The first group is comprised of calcium phosphate, which can be highly diluted (1:20,000 *m*:*v*) and does not need standard addition calibration. The calibration range is from the determination limit of 600 mg L^−1^ in the initial sample (ten times the standard deviation multiplied by the dilution ratio) up to 0.4 mg L^−1^ in solution, which corresponds to 8000 mg L^−1^ of F in the initial sample. The concentration of 0.1 mg L^−1^ of F in solution relates to the threshold value of 2000 mg kg^−1^.

The second group consists of other materials with a threshold F content of 150 mg L^−1^. From the point of view of the decision on the fulfillment of the official requirements f, the sample dilution ratio would be 1500 *m*:*v*, which would result in 0.1 mg L^−1^ concentration in solution, related to the threshold value. The range of the method would be, from the determination limit, i.e., 50 mg kg^−1^ up to 450 mg kg^−1^, which corresponds to 0.3 mg L^−1^ of F in solution (the upper concentration is less than 0.4 mg L^−1^ due to the use of a standard addition calibration, i.e., addition of 0.1 mg L^−1^ of F). However, the results of analyses of large numbers and variable types of animal feed of various origins, executed over a few years using the potentiometric method (over 80 samples—Section 3.1 and Section 3.4), indicate low fluoride concentration, much lower than the threshold value. Therefore, also the HR-CS GFMAS method could be set to determine the extremely low content of fluorine. Example analysis ranges related to dilutions of 1:50 and 1:200 *m*:*v* are presented in Table 4. The detection and determination limits recalculated for the initial sample at the lowest recommended dilution (1:50) are 0.5 and 1.5 mg kg^−1^, respectively.

The developed HR-CS GFMAS method was designed to determine fluorine in a wide range of concentrations in various materials using the same experimental conditions, volumes, and modifiers. The variable parameter is sample dilution. 

The drawback of the method is the contamination of the graphite system with fluorine (coming from the available gallium salt). It is taken into account by subtracting the blank, but it narrows the calibration range of the method. The need for standard addition calibration limits the upper part of the calibration range.

Another drawback of the HR-CS GFMAS method are memory effects, which can be observed as increased GaF signals for pure deionized water measured after solutions with higher F content. The effect should be controlled by periodic measurements of blank.

The relative standard deviation calculated for parallel analyses is at the level of a few percent. For the determination of F in calcium phosphate, repeatability was evaluated. Nine pairs of results were obtained (Table S2; the results of each pair in repeatability conditions and particular pairs possibly in reproducibility conditions). The average result was equal to 1658 mg kg^−1^, the average difference was 11 mg kg^−1^ and the average relative absolute value of differences was 2.5%.

The accuracy of the HR-CS GFMAS method was evaluated in the next section. 

### 3.3. Comparison of ISE and HR-CS GFMAS Methods

The samples S1–S3 were analyzed using ISE and HR-CS GFMAS methods. The results of both methods shown in Table 5 are consistent with each other, which confirms the good accuracy of the methods. No underestimation of ISE results was observed, in contrast to the experiment with added Fe and Al, discussed in Section 3.1. Possibly, the original content of Fe and Al in the investigated samples is too low to cause a decrease in results.

The consistency of the results of the ISE method (analysis immediately after extraction) and the HR-CS GFMAS method (filtrate analysis from the extract at least a few days after extraction) indicates that there is no fluoride in the sediment emerging before HR-CS GFMAS measurement. The consistency of the results of both methods also informs that fluorine in extracts is in the fluoride form (in HR-CS GFMAS, the total amount of fluorine is determined, while in the ISE method only fluoride anions are detected).

As Table 5 shows, the precision of analysis of samples S1–S3 is comparable. The precision can also be compared, taking into account the results of calcium phosphate from Tables S2 and S3. The differences of two parallel results were evaluated. The average relative absolute value of differences for nine pairs of results obtained in reproducibility conditions was small (~3%) and similar for both methods.

A comparison of ISE and HR-CS GFMAS methods is further shown in Table 6 and Table S4.

The HR-CS GFMAS method has the potential to decrease the detection limit. Exemplarily, a higher volume of investigated solution can be injected, and further efforts to find less polluted gallium can be undertaken. 

Undoubtedly, the ISE technique is faster and cheaper. On the other hand, in the HR-CS GFMAS, an autosampler is utilized.

### 3.4. Results of Fluorides Monitoring in Animal Feed Used in Poland in 2021–2023

The ISE method was widely applied for determining fluorides. Table 7 shows the results of fluoride testing in different types of feed samples collected in Poland in the years 2021–2023. Fluoride content in feed materials, with respect to mean values, varied from low content in plant and animal materials, respectively 4.5 mg kg^−1^ and 7.3 mg kg^−1^ to medium content in calcium carbonate (37.2 mg kg^−1^) and high content in feed phosphates (2790 mg kg^−1^). For feed materials of plant and animal origin, the fluoride levels found were 20–30 times lower than the maximum level of 150 mg kg^−1^, without posing a risk to animals.

## 4. Conclusions

It was determined that the calibration relationship in the ISE method is linear in the 0.1–10 mg L^−1^ fluoride concentration range. At the F concentration below 0.1 mg L^−1^ (0.1 mg L^−1^ in solution relates to 40 mg kg^−1^ in a sample), the calibration relationship is nonlinear, and interpolation, instead of the standard addition method, has to be applied for calibration. Model studies indicated the possibility of a certain underestimation of results of fluoride determination by the ISE method in samples with low fluoride content (recovery reduced to 90–80%). It may be due to a change in the slope of the calibration curve by the matrix components or the consumption of a certain amount of fluorides by cations (such as Fe^3+^ or Al^3+^). These matrix effects will not be detected in proficiency testing, as almost all laboratories use the same ISE method. Therefore, it is essential to have an alternative method for comparison.

A new method for determining F in animal feed using a high-resolution continuum graphite furnace molecular absorption spectrometry (HR-CS GFMAS) was developed. The feed samples turned out to be a challenge for the HR-CSGFMAS method. The components of the measured solutions caused chemical interferences (formation of competing molecules of CaF, GaCl, and GaP) and spectral effects (a spectrum appearing in the measurement phase before the GaF signal, a spectrum of a P-containing molecule, and atomic lines, e.g., gallium lines). Other difficulties were the contamination of available gallium compounds with fluorine and memory effects.

The following strategy, undertaken in HR-CS GFMAS, turned out to be effective and enabled to eliminate/diminish interferences as follows:

– Application of a molecule providing the best sensitivity (GaF), which allows for higher dilution of samples.
– Relatively low measurement temperature, 1300 °C, which is sufficient to produce GaF molecules but too low to lead to the formation of interfering compounds.
– Using the standard addition method to eliminate chemical interferences (except for highly diluted samples, where the standard addition method is unnecessary).

Excellent sensitivity was achieved in the HR-CS GFMAS (characteristic mass of 2 pg), better than reported in the literature. 

The quantification limits of ISE and HR-CS GFMAS methods are comparable and satisfactory (1.0 and 1.5 mg kg^−1^, respectively), as well as precision (relative standard deviation at the level of a few percent). The consistency of the results obtained by the ISE and HR-CS GFMAS methods indicates both the absence of matrix effects and the presence of fluorine in extracts in fluoride anion form.

The ISE method has been positively verified in many proficiency testing programs (13 rounds for various materials containing 10–1800 mg kg^−1^ F).

Generally, it was confirmed that the ISE method is simple, fast, and cost-effective and is a good tool for routine control of fluoride content in the feed. The HR-CS GFMAS can be used alternatively. The application of both methods enables their cross-validation. 

The results of extensive ISE tests conducted in Poland in 2021–2023 have shown that in the vast majority of cases, the fluoride content is more than an order of magnitude lower than the threshold values, which proves that animal feed does not pose a risk of excessive fluoride intake by animals or humans consuming this animal food.

## Figures and Tables

**Table 1:** Program of graphite furnace heating.

Step	Step Name	Temperature, °C	Heating Rate, °C s^−1^	Hold Time, s	Argon Flow	Remarks
1	Drying	80	6	15	Max	Injection ^1^
2	Drying	90	3	10	Max	
3	Drying	110	5	10	Max	
4	Pyrolysis	350	20	10	Max	
5	Pd reduction	1100	100	10	Max	
6	Drying	70	NP ^3^	10	Max	
7	Drying	80	2	10	Max	Injection ^2^
8	Drying	105	5	30	Max	
9	Drying	250	50	5	Max	
10	Pyrolysis	500	50	20	Max	
11	Gas adaptation	500	0	5	Stop	
12	Vaporization	1300	1500	5	Stop	
13	Cleaning	2500	500	9	Max	

^1^ First injection took place before step “1” and was 5 µL of a solution containing 0.1% Pd and 0.003% Zr and 6 µL of a solution containing 0.5% of Ga (total volume 11 µL). ^2^ Second injection took place in step “7” and was 6 µL of a sample, 6 µL of a solution containing 0.5% of Ga, and 5 µL of a solution containing 0.4% of NaAc (total volume 17 µL). ^3^ No power.

**Table 2:** Results of interlaboratory comparisons on fluoride determination in animal feed in 2006–2023.

PT Year	Type of Feed	Number of Laboratories/ Number of ISE Results	Assigned Value, mg kg^−1^	Standard Deviation, mg kg^−1^	NLF Result Obtained Using ISE Method, mg kg^−1^	z-Score
2006 a	Feed phosphate	4/4	939	33.9	970	0.82
2006 a	Feed phosphate	4/4	1799	92.5	1956	0.96
2007 a	Feed phosphate	4/4	817	76.6	894	1.02
2011 a	Premixture for layer	4/4	16.1	2.83	18.4	0.82
2011 b	Compound feed for layers	10/9	9.88	5.40	6.18	−0.70
2013 b	Compound feed for chickens	11/10	21.3	4.13	20.7	−0.14
2014 b	Complementary feed for pigs	11/10	18.8	3.42	17.8	−0.30
2015 b	Compound feed for poultry	13/11	22.0	4.49	22.5	0.10
2016 b	Complementary feed for pigs	12/10	18.3	3.42	18.5	0.10
2017 b	Complementary feed for piglets	11/9	28.0	1.93	30.7	1.40
2020 b	Compound feed for broilers	8/6	15.0	2.61	17.0	0.76
2022 b	Compound feed for piglets	10/7	17.0	3.98	15.4	−0.40
2023 b	Fortified feed for poultry	8/6	75.8	14.2	69.6	−0.43

a—interlaboratory comparisons organized by the National Laboratory for Feedingstuffs (NLF, Lublin, Poland) as a part of the tasks of the reference laboratory; b—international proficiency test organized by the Österreichische Agentur für Gesundheit und Ernährungssicherheit International Analytical Group (AGES-IAG-Feedingstuffs, Vienna, Austria).

**Table 3:** Figures of merit.

Parameter	Value of Parameter
Characteristic concentration, pg	2.1
Calibration curve parameters:	
– equation (range up to 0.4 mg L^−1^)	y = 12.831x + 0.122
– determination coefficient, R^2^	0.9959
– correlation coefficient, R	0.9980
Blank (H_2_O):	
– average F concentration, mg L^−1^	0.030
– standard deviation (s), mg L^−1^	0.0032
– instrumental detection limit (3 s), mg L^−1^	0.0096

**Table 4:** Dilution of samples and calibration range.

Analytical Parameter	Calcium Phosphate	Other Feed
Threshold F concentration, mg kg^−1^	2000	150
Dilution from the threshold content of F in the sample to 0.1 mg L^−1^ of F in the measured solution	1:20,000, *m*:*v* (1.25 g/50 mL → 1:10 → 1:10 → 1:5)	1:1500, *m*:*v* (1.25 g/50 mL → 1:5 → 1:7.5)
Parameters recalculated for sample:		
– blank	600	45
– detection limit, mg kg^−1^	200	15
– calibration range, mg kg^−1^	600–8000	50–450
Dilution	-	1:200, *m*:*v* (1.25 g/50 mL → 1:5)
Parameters recalculated for sample:		
– blank		6
– detection limit, mg kg^−1^		2
– calibration range, mg kg^−1^		6–60
Dilution	-	1:50, *m*:*v* (1.25 g/50 mL → 4:5)
Parameters recalculated for sample:		
– blank		1.5
– detection limit, mg kg^−1^		0.5
– calibration range, mg kg^−1^		1.5–15

**Table 5:** Results of fluorine determination, mg kg^−1^. Analyses using both techniques were performed in different laboratories at an interval of at least a few days.

Sample	Proficiency Testing ^1^	ISE		HR-CS GFMAS	
	Assigned Value ± Standard Deviation	Average Result ± Standard Deviation (Number of Parallel Analyses)	RSD, %	Average Result ± Standard Deviation (Number of Parallel Analyses)	RSD, %
S1 Calcium phosphate	-	1680 ± 43 (8)	2.6	1690 ± 50 (8)	2.9
S2 Mixed feed for turkey	11.2 ± 3.7	13.1 ± 1.3 (2)	9.9	12.5 ± 0.8 (3)	6.4
S3 Spiked feed for chicken	70 ± 26	64 ± 6 (2)	9.3	66 ± 7 (3)	9.8

^1^ Proficiency testing organized by AGES-IAG-Feedingstuffs (Vienna, Austria).

**Table 6:** Comparison of features of ISE and HR-CS GFMAS methods (relative evaluation: “+” positive, “−“negative, “+/−“ neutral).

Evaluated Parameter	ISE	HR-CS GFMAS
Sensitivity	+	+
Detectability	+/−	+
Precision	+	+
Total amount of F determination	−	+
Interferences: Ca/PO_4_^3−^ effect	+	+/−
Interferences: Fe, Al effect	+/−	+
Interferences: HCl effect	+	−
Calibration range and parameters	+	+/−
Investment costs	+	−
Running costs	+	−
Speed of analysis	+	−
Automation *	−	+
Contamination risk	+	−

* Autosampler utilization.

**Table 7:** Fluoride content in different types of animal feed samples collected in Poland in the years 2021–2023.

Kind of Animal Feed (Number of Samples of This Type)	Content of Fluorides, mg kg^−1^	Mean Value, mg kg^−1^	Median, mg kg^−1^	Standard Deviation, mg kg^−1^	Coefficient of Variation, %	Threshold Value, mg kg^−1^
Feed materials of plant origin (34)	2.3–8.9	4.5	4.4	1.4	31	150
Feed materials of animal origin (18)	2.8–25.3	7.3	4.7	6.1	84	150
Calcium carbonate (13)	9.7–73.1	37.2	36.4	19.1	51	350
Calcium phosphate (5)	1620–4420	2790	2230	1250	45	2000
Compound feed for poultry (17)	5.2–20.7	12.0	11.5	4.7	39	150

## Data Availability

The raw data supporting the conclusions of this article will be made available by the authors on request.

## Supplementary Materials

The following supporting information can be downloaded at: https://www.mdpi.com/article/10.3390/ma17122812/s1. Figure S1: Example calibration curve in ISE method The curve is entirely linear in the range 0.1 mg L^−1^–10 mg L^−1^ (R^2^= 1.0000). It starts to curve at the concentration of 0.1 mg L^−1^. When the whole range of F concentration, 0.01–10 mg L^−1^, R^2^= 0.9925 for linear approximation and R^2^= 0.9988 for quadratic approximation; Figure S2: Signals of a solution of sample S1 (dilution 1:600 *m*:*v*, 0.3 M HCl), obtained using the following pyrolysis/vaporization temperatures: (a) 300 °C/1300 °C, (b) 900 °C/1300 °C, (c) 500 °C/1000 °C, (d) 500 °C/1300 °C, (e) 500 °C/1600 °C. The main figure of each subfigure presents absorbance-time changes (the blue line is the analyte line, and the red line is the background line). The smaller figure of each subfigure denominates enlarged wavelength-resolved absorption spectrum. The vertical orange line denominates the central pixel of the number 101; Figure S3: Signals of a solution of sample S3 (dilution 1:600 *m*:*v*), obtained using the following pyrolysis/vaporization temperatures: (a) 300 °C/1300 °C, (b) 900 °C/1300 °C, (c) 500 °C/1000 °C, (d) 500 °C/1300 °C, (e) 500 °C/1600 °C. The main figure of each subfigure presents absorbance-time changes (the blue line is the analyte line, and the red line is the background line). The smaller figure of each subfigure denominates enlarged wavelength-resolved absorption spectrum. The vertical orange line denominates the central pixel of the number 101; Figure S4: Signals of a 0.1 mg L^-1^ solution of fluorine standard as NaF in 1 M HCl, obtained using the following pyrolysis/vaporization temperatures: (a) 300 °C/1300 °C, (b) 900 °C/1300 °C, (c) 500 °C/1000 °C, (d) 500 °C/1300 °C, (e) 500 °C/1600 °C. The main figure of each subfigure presents absorbance-time changes (the blue line is the analyte line, and the red line is the background line). The smaller figure of each subfigure denominates an enlarged wavelength-resolved absorption spectrum. The vertical orange line denominates the central pixel of the number 101; Table S1: Evaluation of intralaboratory-reproducibility of analysis of samples with low F content using ISE method; Table S2: Evaluation of intralaboratory-reproducibility of analysis of calcium phosphate using ISE method; Table S3: Evaluation of intralaboratory-reproducibility of analysis of calcium phosphate using HR-CS GFMAS method. Samples dilution 1:20,000, *m*:*v*; Table S4: Summary of HR-CS GFMAS and ISE methods.
