# Supplementary material for: Determination of Fluorine by Ion-Selective Electrode and High-Resolution Continuum Source Graphite Furnace Molecular Absorption Spectrometry with Respect to Animal Feed Safety
Source: Materials (Basel). 2024 Jun 9;17(12):2812. doi: 10.3390/ma17122812 (PMC11204728; doi:10.3390/ma17122812)
Supplement: Supplementary file 1 [file materials-17-02812-s001.zip › materials-2966766-supplementary.pdf]

materials

Supplementary material

Determination of Fluorine by Ion-Selective Electrode and High-Resolution Continuum Source Graphite Furnace Molecular Absorption Spectrometry with Respect to Animal Feed Safety

Zofia Kowalewska [1,*], Karolina Goluch [2], Waldemar Korol [2], Rafał Olchowski [3] and Ryszard Dobrowolski [4,*]

[1] Faculty of Civil Engineering, Mechanics and Petrochemistry, Warsaw University of Technology, Łukasiewicza 17, 09-410 Plock, Poland
[2] National Laboratory for Feedingstuffs, National Research Institute of Animal Production, Chmielna 2 Str., 20-079 Lublin, Poland; karolina.goluch@iz.edu.pl (K.G.); korol@clpp.lublin.pl (W.K.)
[3] Department of Pharmacology, Toxicology and Environmental Protection, Faculty of Veterinary Medicine, University of Life Sciences, Akademicka Sq. 12, 20-950 Lublin, Poland; rafal.olchowski@up.lublin.pl
[4] Department of Analytical Chemistry, Institute of Chemical Sciences, Faculty of Chemistry, Maria Curie-Skłodowska University, M. C. Skłodowska Sq. 3, 20-031 Lublin, Poland
* Correspondence: zofia.kowalewska@pw.edu.pl (Z.K.); rdobrow@poczta.umcs.lublin.pl (R.D.).

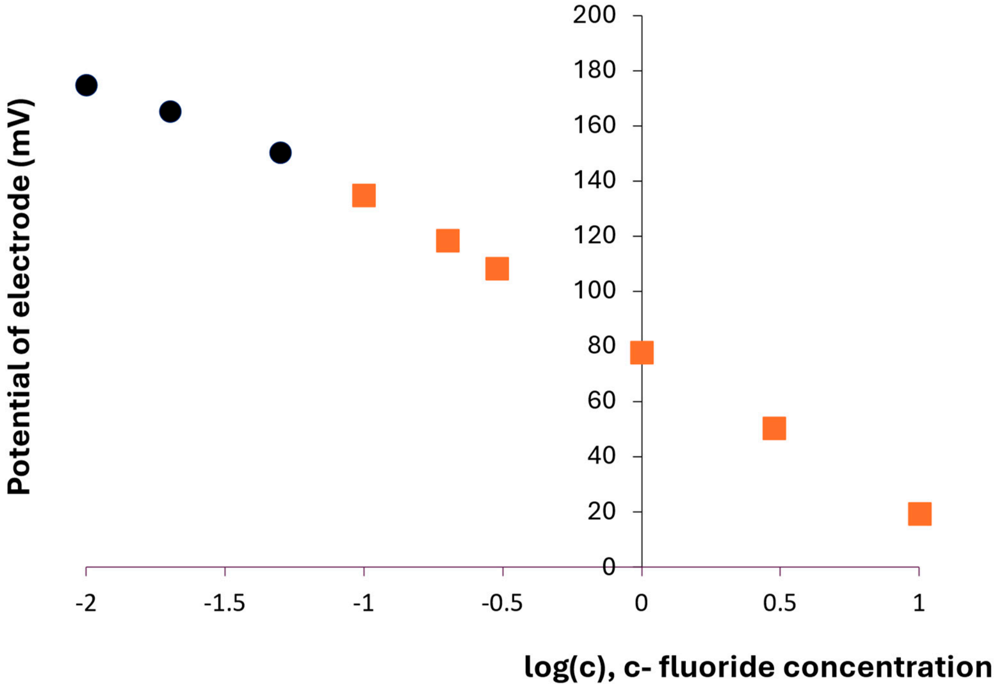

Figure S1. Example calibration curve in ISE method The curve is entirely linear in the range 0.1 mg L^{-1} - 10 mg L^{-1} (R^2= 1.0000). It starts to curve at the concentration of 0.1 mg L^{-1}. When the whole range of F concentration, 0.01-10 mg L^{-1}, R^2= 0.9925 for linear approximation and R^2= 0.9988 for quadratic approximation.

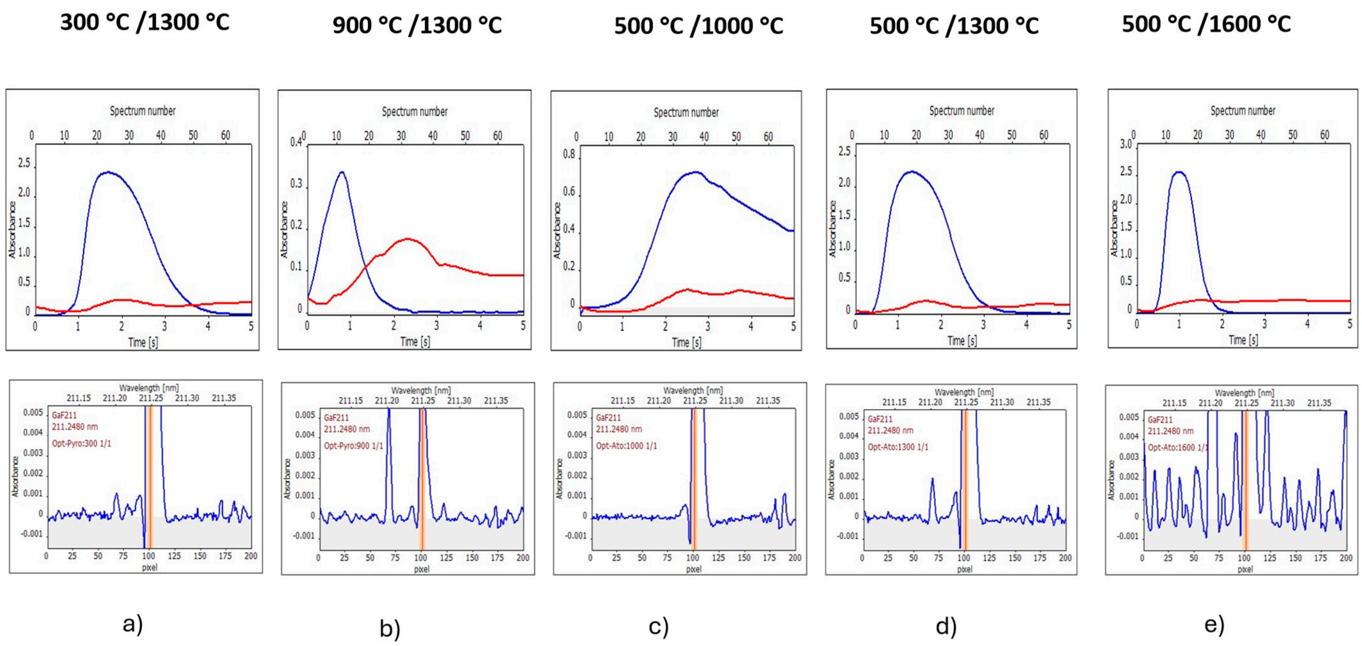

Figure S2. Signals of a solution of sample S1 (dilution 1:600 m:v, 0.3 M HCl), obtained using the following pyrolysis/vaporization temperatures: (a) 300 °C /1300 °C, (b) 900 °C /1300 °C, (c) 500 °C /1000 °C, (d) 500 °C /1300 °C, (e) 500 °C /1600 °C. The main figure of each subfigure presents absorbance-time changes (the blue line is the analyte line, and the red line is the background line). The smaller figure of each subfigure denominates enlarged wavelength-resolved absorption spectrum. The vertical orange line denominates the central pixel of the number 101.

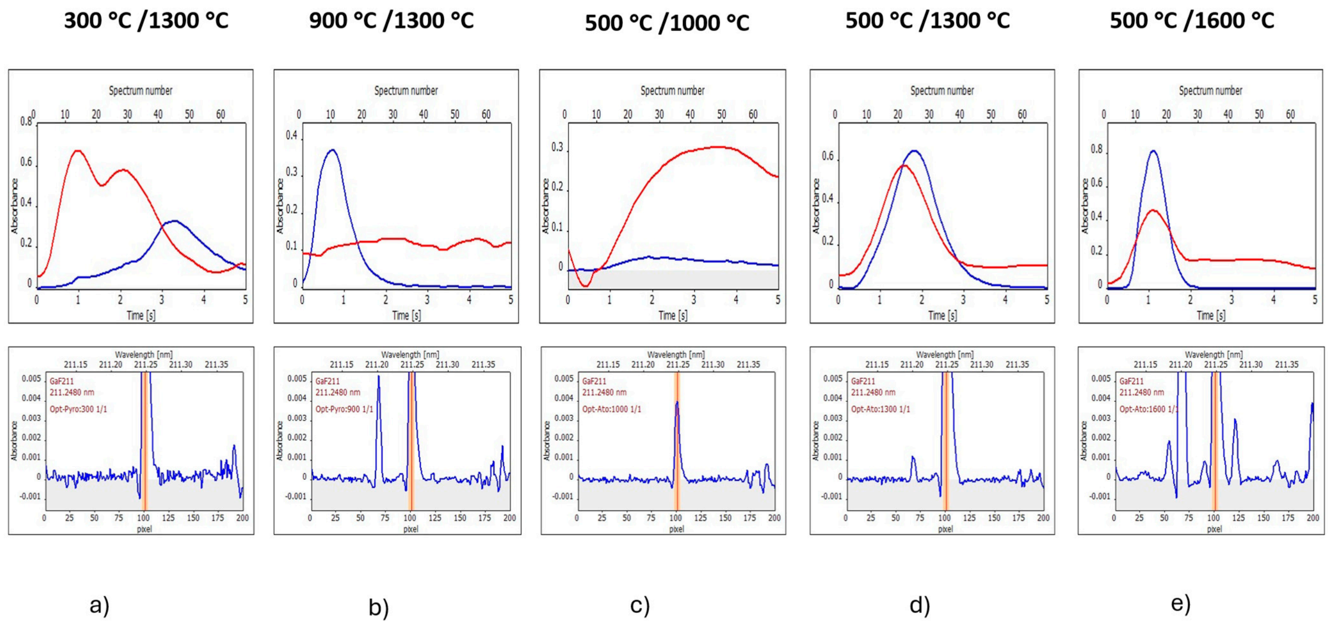

Figure S3. Signals of a solution of sample S3 (dilution 1:600 m:v), obtained using the following pyrolysis/vaporization temperatures: (a) 300 °C /1300 °C, (b) 900 °C /1300 °C, (c) 500 °C /1000 °C, (d) 500 °C /1300 °C, (e) 500 °C /1600 °C. The main figure of each subfigure presents absorbance-time changes (the blue line is the analyte line, and the red line is the background line). The smaller figure of each subfigure denominates enlarged wavelength-resolved absorption spectrum. The vertical orange line denominates the central pixel of the number 101.

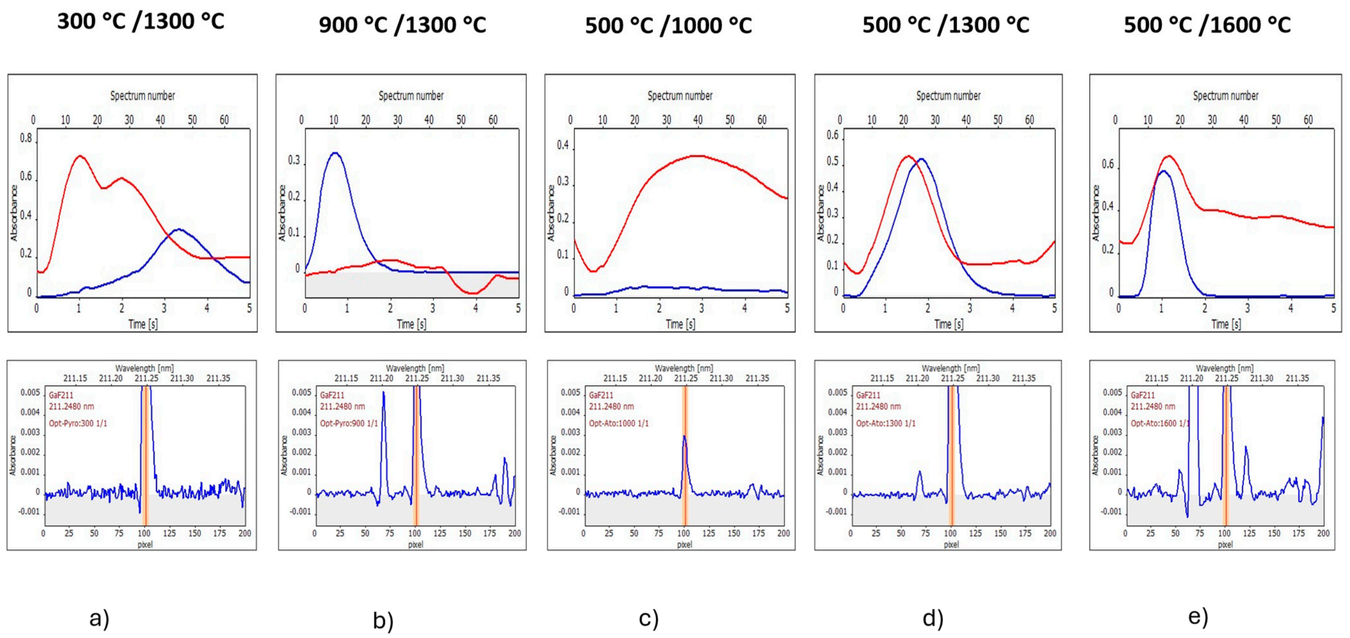

Figure S4. Signals of a 0.1 mg L-1 solution of fluorine standard as NaF in 1M HCl, obtained using the following pyrolysis/vaporization temperatures: (a) 300 °C /1300 °C, (b) 900 °C /1300 °C, (c) 500 °C /1000 °C, (d) 500 °C /1300 °C, (e) 500 °C /1600 °C. The main figure of each subfigure presents absorbance-time changes (the blue line is the analyte line, and the red line is the background line). The smaller figure of each subfigure denominates an enlarged wavelength-resolved absorption spectrum. The vertical orange line denominates the central pixel of the number 101.

Table S1. Evaluation of intralaboratory-reproducibility of analysis of samples with low F content using ISE method.

Pair number	Result 1, mg kg-1	Result 2, mg kg-1	Average result, mg kg-1	Difference of resutls, mg kg-1	Relative absolute value of difference, %
1	5.33	5.24	5.29	0.09	1.7
2	5,52	5.79	5.66	-0.27	4.8
3	5,43	5.19	5.31	0.24	4.5
4	5.13	5.32	5.23	-0.19	3.6
5	5.09	4.87	4.98	0.22	4.4
6	6.07	5.80	5.94	0.27	4.6
7	5.20	4.99	5.10	0.21	4.1
8	5.96	6.18	6.07	-0.22	3.6
9	5.30	5.51	5.41	-0.21	3.9
10	5.41	5.21	5.31	0.20	3.8
11	5.21	5.12	5.17	0.09	1.7
		Average	5.40	0.04	3.7

Results 1 and 2 were always obtained in repeatibility conditions and particular pairs of results could be obtained in various days and in changed conditions.

Table S2. Evaluation of intralaboratory-reproducibility of analysis of calcium phosphate using ISE method.

Pair number	Result 1, mg kg-1	Result 2, mg kg-1	Average result, mg kg-1	Difference of resutls, mg kg-1	Relative absolute value of difference, %
1	4329	4353	4353	-24	0.6

2	3738	3892	3892	-154	4.0
3	1626	1622	1622	4	0.3
4	1894	1835	1835	59	3.2
5	2207	2258	2258	-51	2.7
6	1482	1517	1517	-35	2.3
7	1736	1862	1862	-126	6.8
8	1698	1672	1672	26	1.6
9	1396	1348	1348	48	3.6
		Average	2262	-28	2.9

Results 1 and 2 were always obtained in repeatibility conditions and particular pairs of results could be obtained in various days and in changed conditions.

Table S3. Evaluation of intralaboratory-reproducibility of analysis of calcium phosphate using HR-CS GFMAS method. Samples dilution 1 : 20 000, m:v**.**

Pair number	Result 1, mg kg^{-1}	Result 2, mg kg^{-1}	Average result, mg kg^{-1}	Difference of resutls, mg kg^{-1}	Relative absolute value of difference, %
1	1680	1644	1662	-36	2.2
2	1528	1539	1533	11	0.7
3	1561	1628	1595	67	4.2
4	1723	1720	1722	-3	0.2
5	1708	1619	1663	-89	5.4
6	1781	1721	1751	-60	3.4
7	1634	1657	1646	23	1.4
8	1680	1598	1689	18	1.1
9	1672	1644	1658	-28	1.7
		Average	1658	-11	2.5

Results 1 and 2 were always obtained in repeatibility conditions and particular pairs of results could be obtained in various days.

Table S4. Summary of HR-CS GFMAS and ISE methods.

HR-CS GFMAS	ISE	Evaluated parameter
0.5	0.3	Limit of detection, mg kg^{-1}
1.5	1.0	Limit of quantification, mg kg^{-1}
A few	A few	Precision (relative standard deviation), %
In the case of the threshold value of 150 mg kg^{-1}:<br>-1:50 m:v: (1.5-15 mg kg^{-1} of F)<br>-1:200 m:v: (6-60 mg kg^{-1} of F)<br>-1:1500 m:v (50-450 mg kg^{-1} of F)<br><br>In the case of the threshold value of 2000 mg kg^{-1}:<br>-1:20 000 m:v (600-8000 mg kg^{-1} of F)	For all samples and threshold values: 1:400 m:v	Samples dilution

-Calcium phosphate (1:20 000 m:v dilution): calibration curve<br>-Other feed: standard addition calibration	Calibration relationship:<br>-non-linear (0.01-0.1 mg L^{-1} of fluorides in solution, i.e. 4-40 mg kg^{-1}) – interpolation for calibration<br>-linear (0.1-10 mg L^{-1} of fluorides in solution; i.e. 40-4000 mg kg^{-1}) – standard addition calibration	Calibration way
